# Changing Patterns of Health in Communities Impacted by a Bioenergy Project in Northern Sierra Leone

**DOI:** 10.3390/ijerph111212997

**Published:** 2014-12-12

**Authors:** Astrid M. Knoblauch, Mary H. Hodges, Mohamed S. Bah, Habib I. Kamara, Anita Kargbo, Jusufu Paye, Hamid Turay, Emmanuel D. Nyorkor, Mark J. Divall, Yaobi Zhang, Jürg Utzinger, Mirko S. Winkler

**Affiliations:** 1 Department of Epidemiology and Public Health, Swiss Tropical and Public Health Institute, P.O. Box, CH-4002 Basel, Switzerland; E-Mails: astrid.knoblauch@unibas.ch (A.M.K.); juerg.utzinger@unibas.ch (J.U.); 2 University of Basel, P.O. Box, CH-4003 Basel, Switzerland; 3 Helen Keller International Sierra Leone, P.O. Box, Freetown, Sierra Leone; E-Mails: mhodges@hki.org (M.H.H.); mdbah@hki.org (M.S.B.); hkamara@hki.org (H.I.K.); akargbo@hki.org (A.K.); jpaye@hki.org (J.P.); thamid@hki.org (H.T.); enyorkor@hki.org (E.D.N.); 4 SHAPE Consulting Ltd., Pretoria 0062, South Africa; E-Mail: mdivall@shapeconsulting.org; 5 Helen Keller International, Regional Office for Africa, P.O. Box, Dakar, Senegal; E-Mail: yzhang@hki.org

**Keywords:** anemia, bioenergy, health impact assessment, helminth infection, malaria, nutritional status, Sierra Leone

## Abstract

Large private sector investments in low- and middle-income countries are often critically evaluated with regards to their environmental, social, human rights, and health impacts. A health impact assessment, including a baseline health survey, was commissioned by the Addax Bioenergy Sierra Leone project in 2010. As part of the monitoring, a follow-up survey was conducted three years later. A set of health indicators was assessed at six impacted and two control sites. Most of these indices improved, particularly at the impacted sites. The prevalences of stunting, wasting, and *Plasmodium falciparum* in children under five years of age decreased significantly at impacted sites (all *p* < 0.05) and non-significantly at control sites. Anemia in children and in women of reproductive age (15–49 years) decreased significantly at impacted and control sites (*p* < 0.05 and *p* < 0.001, respectively). Health facility-based deliveries increased significantly at the impacted sites (*p* < 0.05). The prevalences of helminth infections in children aged 10–15 years remained approximately at the same levels, although focal increases at the impacted sites were noted. Access to improved sanitation decreased significantly (*p* < 0.05) at control and non-significantly at impacted sites. Water quality remained poor without significant changes. The epidemiologic monitoring of a bioenergy project provides a useful contribution for evidence-based decision-making.

## 1. Introduction

Large private sector investments in low- and middle-income countries, including agricultural, water resources development and management, and extractive industry projects, are increasingly being developed in remote areas, often associated with vulnerable communities and, thus, subject to international scrutiny [1,2]. The discussion revolves around potential project-related impacts on the environment, people’s health, social cohesion, and human rights [3,4,5]. Proponents and opponents have different views on the extent of positive and negative impacts [6,7]. Potential positive impacts include improved public infrastructure, capacity building, socioeconomic benefits, and better health [8,9]. Potential negative impacts may involve loss of land, environmental degradation, disruption of social cohesion, and widening of wealth disparities [2,10,11,12].

The development and operation of the Addax Bioenergy Sierra Leone (ABSL) ethanol and power project is an example that highlights these challenges and opportunities [13,14,15]. Located near Makeni in Northern Sierra Leone, the ABSL project holds a land lease of 14,300 ha, which is used as a sugarcane plantation to produce an estimated 85,000 m^3^ of ethanol annually, which is to be used for export and the local market [16]. By 2016, sugarcane residual processing will produce 32 MW of electricity per year, of which 15 MW are to be fed into the national grid, contributing 20% of the national requirements.

As of May, 2014, 2750 people had signed either permanent (~40%) or temporary (~60%) work contracts with ABSL, of which 12% were female and 53% originated within a 20-km radius of the ABSL project [16,17]. Given the size of the ABSL project and the importance for the region, it sparked discussions on issues like large land leasing (also referred to as “land grabbing”) and associated negative consequences, such as reduction in food security and deterioration of local livelihoods [2,13,14,15]. Health issues are also relevant, as a project of this magnitude is likely to influence people’s health through a complex interaction of proximal (e.g., altered ecosystems), distal (e.g., transport routes), and cumulative factors (e.g., employment-seeking migration, the reduction of biodiversity due to altered ecosystems, road construction, and accidents) [3,18,19].

**Figure 1:**
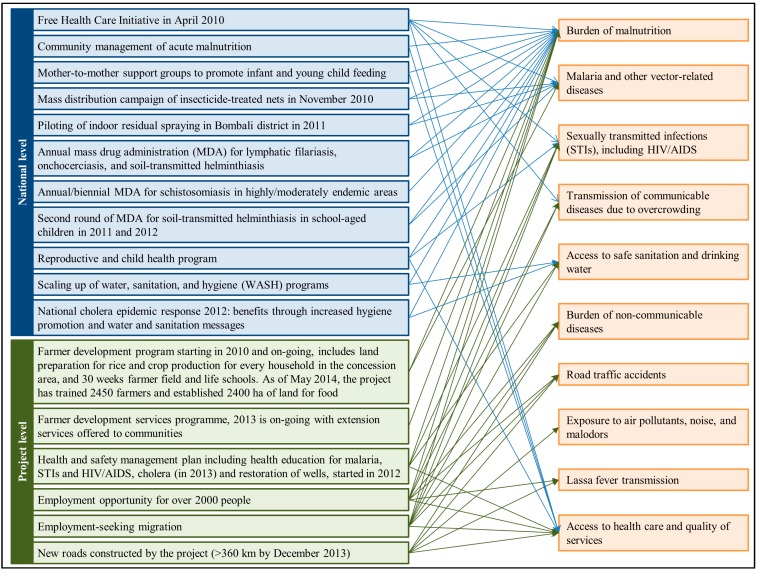
National interventions or programs and interventions initiated by the Addax Bioenergy Sierra Leone (ABSL) project between 2010 and 2013 and the associated health impacts.

During the feasibility phase of the ABSL project between 2008 and 2010, the group of development finance institutions chosen to fund part of the ABSL project commissioned the company to conduct an environmental, social, and health impact assessment (ESHIA) in compliance with the International Finance Corporation’s (IFC) performance standards [16,20,21]. Within the ESHIA, ABSL commissioned a cross-sectional baseline health survey (BHS) to determine health conditions in communities that could potentially be affected by the project. This BHS was conducted in December 2010, prior to the commencement of the main construction activities [22]. Potential health impacts related to the ABSL project have been described elsewhere [20,22]. Since 2010, numerous public health interventions have been implemented by the Sierra Leonean health sector [23,24,25,26]. Additionally, community and infrastructural developments within the area have been launched by the ABSL project. The characteristics of these interventions and their potential impact on health are conceptualized in Figure 1.

In order to monitor changing patterns of people’s health and to determine the potential health impacts of ABSL activities, the HIA recommended ABSL to conduct cross-sectional health surveys once every three years as part of the environmental and social monitoring. Here, we report findings from the first follow-up health survey, conducted three years after the initial BHS and implemented at the same sentinel sites [22]. A range of health indicators serve for comparison of the baseline status with the three-year follow-up. Moreover, findings at sentinel sites impacted by the ABSL project are compared with findings at control sentinel sites. The methodology employed is discussed in the context of current practice in health impact assessment (HIA).

## 2. Materials and Methods

### 2.1. Ethical Considerations

Approval for the study was provided by the Sierra Leone Ethics and Scientific Review Committee of the Ministry of Health and Sanitation (MoHS). Community sensitization was performed through meetings with leaders followed by informed consent (signed or fingerprinted) obtained from participating heads of households and mothers/caregivers. For the school survey, written informed consent was given by teachers, whilst children assented orally. Children found with mild or moderate anemia (hemoglobin (Hb) 7–11.0 g/dL) or severe anemia (Hb < 7 g/dL) were provided with iron supplements or referred to a regional hospital for further investigation, respectively. Artemisinin-based combination therapy (ACT), using artesunate-amodiaquine, was given to children who were found positive for *Plasmodium falciparum*. Children found with concurrent *P. falciparum* infection and anemia were treated with the required ACT dosage, followed by iron supplements. Moderately to severely anemic women were counseled and referred to a health facility if indicated based on their clinical condition. School-age children with confirmed *Schistosoma* infection received praziquantel (40 mg/kg). Treatment was administered by the community health officer in collaboration with the MoHS peripheral health unit (PHU) staff. In adherence to MoHS regulations, all treatments were given free of charge and provided by the PHU, which recorded the identity of recipients and outgoing stock.

### 2.2. Study Area

**Figure 2:**
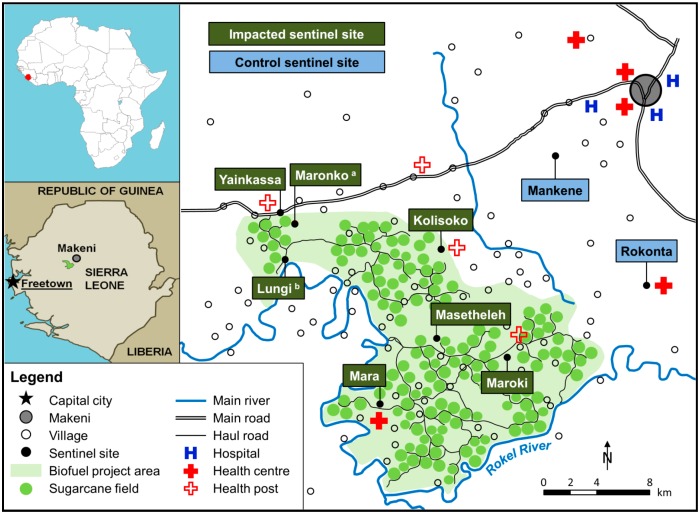
Sentinel sites of the follow-up health survey in the ABSL project study area in Northern Sierra Leone, 2013.

The ABSL project is located in the Northern region of Sierra Leone in Bombali and Tonkolili districts, west of Makeni town [16]. The project area uses about 10,000 ha for sugarcane plantation, and an additional 4300 ha has been designated for infrastructural build-up, farming fields, and ecological conservation areas. An estimated 30,000 people reside within the project area, which is limited by the Lunsar-Makeni highway to the north and by the Rokel River to the south and west (Figure 2) [27].

### 2.3. Study Design and Sampling Methodology

The three-year follow-up survey used the same modular, cross-sectional study design and sentinel sites as the BHS [22,28]. The sentinel site selection procedure took into consideration: (i) the prevailing eco-epidemiologic characteristics in the study area; (ii) the exposure to ABSL project activities (e.g., infrastructural developments or community development initiatives); and (iii) the presence of health facilities. These criteria led to the selection of six sentinel sites within the ABSL project area (designated as impacted sites; Yainkassa, Maronko, Kolisoko, Masetheleh, Maroki, and Mara) and two sentinel sites outside the project area, serving as controls (Mankene and Rokonta; Figure 2). Four sites had a health facility. Of note, the two control sites are within 10 km of the ABSL project, but not directly impacted by its activities (e.g., road developments, employment). In order to control for the effect of seasonal fluctuations on specific health conditions (e.g., malaria and wasting), the two surveys, spaced three years apart, were both carried out in mid-December.

Within these sites, households served as the primary sampling units. Four study interviewers selected households using a random sampling procedure for the first household, followed by proximity sampling (i.e., next-door household) [28]. The inclusion criterion for a household was the presence of a mother with at least one child under five years of age.

Mothers/caregivers were invited to respond to a pre-tested questionnaire investigating sociodemographic and health issues. After completion of the questionnaire, all household members were invited to visit a clinical field unit located at a central place within the sites. Children under five years of age had their weight (digital scale, Seca 877; Hamburg, Germany) and their height measured (portable stadiometer, Seca 213; Hamburg, Germany) [29]. Following a capillary blood sample from a finger-prick, children aged 6–59 months were tested for *P*. *falciparum* infection using a rapid diagnostic test (RDT; First Response^®^, Premier Medical Corporation Ltd; Nani Daman, India) [30]. Additionally, Hb concentration was determined using a HemoCue^®^ 201+ testing device (HemoCue Hb 201 System; HemoCue AB, Ängelholm, Sweden) [31]. Females aged 15–49 years also provided a finger-prick sample to determine Hb concentration, and their pregnancy status was recorded by the clinical health officer based on verbal reporting. For quality control, clinical data were checked for completeness and entered into a database directly in the field. Inconsistencies or incomplete information were managed and corrected by returning to the respective households.

School-going children aged 10–15 years were randomly selected from schools serving the selected sentinel sites to assess the prevalence and intensity of soil-transmitted helminths and *Schistosoma* infections. There is no school in Maronko, so children in Lungi school serving this site were sampled. At each school, fresh stool and mid-morning urine samples were collected from 30 children; 15 boys and 15 girls. One stool sample per child was examined by microscopy, using the standard Kato–Katz technique [32], to assess the prevalence of infection for *Schistosoma* *mansoni*, *Ascaris* *lumbricoides*, hookworm, and *Trichuris* *trichiura*. *Schistosoma* *haematobium* was assessed in urine samples using the centrifugation method [33].

A water quality analysis was carried out to assess the presence/absence of coliform bacteria as an indicator organism for fecal contamination. By means of sterile 100-mL bottles, one drinking water sample was collected in 10 randomly selected participating households and all drinking water collection points in each sentinel site, in both the BHS and the follow-up survey. In the BHS, the presence/absence of coliform bacteria and *Escherichia coli* were determined, using a Colitag^TM^ water test (CPI International; Santa Rosa, CA, USA) [34]. In the 2013 follow-up survey, the presence/absence and quantity of coliform bacteria were determined using the DelAgua^®^ portable water quality testing kit (DelAgua Water Testing Limited; Marlborough, UK) [35].

### 2.4. Statistical Analysis

Data were recorded directly in the field using EpiData version 3.1 (EpiData Association; Odense, Denmark) and Excel (Microsoft Office; Redmond, DC, USA). Statistical analysis of the biomedical and questionnaire survey data were carried out in STATA (Stata Corp LP; College Station, TX, USA). Data obtained from biomedical samples were adjusted for intra-cluster correlation at the household level, using a generalized estimating equation model, since multiple individuals from the same household participated. Anemia was defined as Hb <11 g/dL in children and pregnant women and as Hb <12 g/dL in non-pregnant women, according to WHO guidelines [36,37]. Two indicators from the questionnaire survey were evaluated: (i) “place of delivery of last born child”; and (ii) “household access to improved sanitation”. Standard categories were used in accordance with the Demographic and Health Survey (DHS) Sierra Leone [38].

Anthropometric data were analyzed using WHO Anthro version 3.2.2 (WHO; Geneva, Switzerland). Wasting (weight-for-height), stunting (height-for-age), and being underweight (weight-for-age) were defined as <−2 standard deviation (SD) scores with reference to the WHO standard population [39]. Water quality was categorized into “absence of coliforms” (0 coliforms/100 mL) and “presence of coliforms” (>0 coliforms/100 mL).

Comparisons between the BHS and the three-year follow-up survey, stratified by impacted and control sites, were made using the χ^2^ test of proportions, including a 95% confidence interval (CI). A *p*-value <0.05 was interpreted as a significant difference.

## 3. Results

### 3.1. Study Compliance

**Figure 3:**
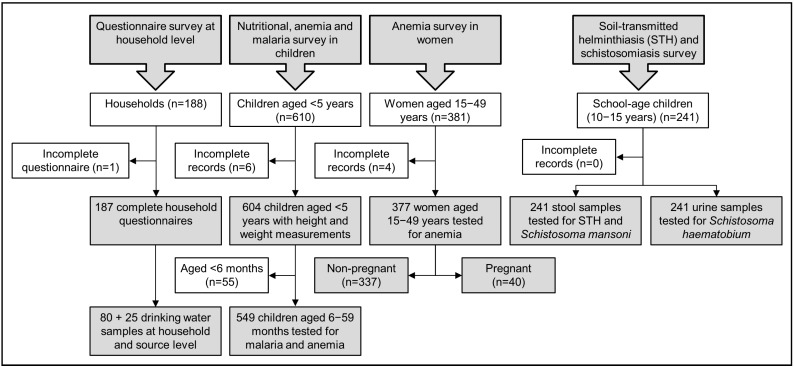
Participation and adherence in the different survey arms of the three-year follow-up health survey in the ABSL project study area, 2013.

Overall, 187 households participated in the follow-up health survey; 137 at the impacted and 50 at the control sites. A total of 1222 individuals (604 children under five years of age, 241 children aged 10–15 years, and 377 women of reproductive age) provided biomedical samples. Drinking water samples were collected and analyzed for a total of 80 households and 25 sources. Figure 3 shows the study compliance during the follow-up survey. Table 1 summarizes sample demographics for the BHS and the three-year follow-up survey, stratified by impacted and control sites.

**Table 1:** Sample sizes at the impacted and the control sentinel sites, in the ABSL project study area, 2010 and 2013.

Characteristics	Impacted Sentinel Sites		Control Sentinel Sites	
	2010	2013	2010	2013
Sentinel sites	6	6	2	2
Households	143	137	51	50
Children aged <59 months	421	390	164	214
Children aged 6–59 months	409	350	161	199
School-going children aged 10–15 years	180	181	60	60
Women aged 15–49 years	284	255	113	122
Non-pregnant	234	230	97	107
Pregnant	50	25	16	15

### 3.2. Anthropometric Indicators in Children Aged <59 Months

**Figure 4:**
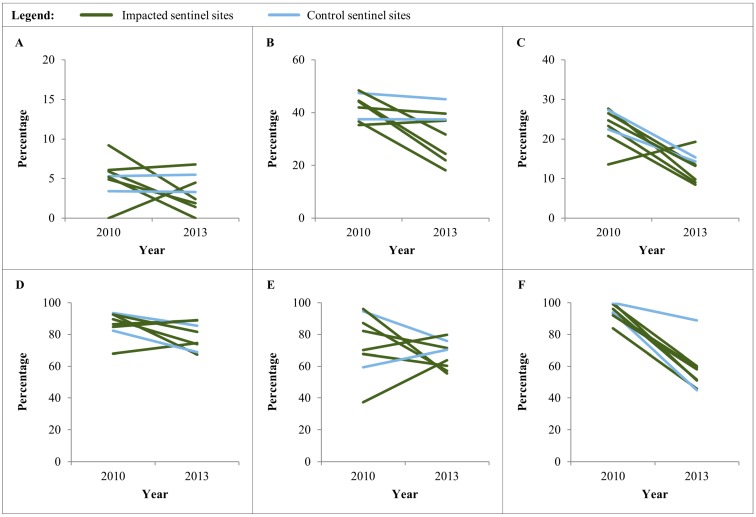
Indicators in children under five years of age in the ABSL study area, stratified by sentinel site, 2010 and 2013. (**A**) Prevalence of wasting (<−2 SD) in children aged <59 months; (**B**) prevalence of stunting (<−2 SD) in children aged <59 months; (**C**) prevalence of being underweight (<−2 SD) in children aged <59 months; (**D**) prevalence of anemia in children aged 6–59 months; (**E**) prevalence of *Plasmodium falciparum* in children aged 6–59 months; and (**F**) insecticide treated net use in the night preceding the survey in children aged 6–59 months.

As shown in Table 2 and Figure 4A–C, the prevalences of wasting, stunting, and being underweight have decreased over the three-year period at the sentinel sites. Wasting decreased significantly in the impacted sites from 5.7% to 2.8% (*p* = 0.044), while remaining stable at the control sites (4.2% in 2010 and 4.3% in 2013; *p* = 0.976). Stunting decreased significantly at the impacted sites (41.8% in 2010 and 32.1% in 2013; *p* = 0.004) and non-significantly at the control sites (42.1% in 2010 and 40.7% in 2013; *p* = 0.781). Being underweight decreased significantly from 23.0% to 13.3% at the impacted and from 25.0% to 15.0% at the control sites (both *p* < 0.001).

**Table 2:** Key health indicators at the impacted and the control sentinel sites, in the ABSL project study area, 2010 and 2013.

		Impacted Sentinel Sites		Control Sentinel Sites	
		2010	2013	2010	2013
**Indicators in children aged <59 months**					
**Sample size (*n*)**	Individuals	421	390	164	214
	Households	149	131	52	50
	Sentinel sites	6	6	2	2
	Mean/sentinel site (range)	68 (24–99)	58 (21–91)	81 (75–86)	100 (83–116)
**Wasting**	Prevalence (95% CI)	5.7 (3.4–8.0)	2.8 (1.0–4.6)	4.3 (0.9–7.7)	4.2 (1.3–7.1)
	Range	0.0–9.2	0.0–6.8	3.4–5.3	3.3–5.5
	*p*-value		0.044		0.976
**Stunting**	Prevalence (95% CI)	41.8 (37.0–46.6)	32.1 (27.3–36.8)	42.1 (34.2–49.9)	40.7 (33.8–47.5)
	Range	35.3–48.5	18.2–39.6	37.5–47.4	37.4–45.1
	*p*-value		0.004		0.781
**Underweight**	Prevalence (95% CI)	23.0 (18.9–27.2)	13.3 (9.8–16.8)	25.0 (18.1–31.9)	15.0 (9.9–20.0)
	Range	13.6–27.7	8.5–19.3	22.4–27.3	14.3–15.4
	*p*-value		<0.001		0.014
**Indicators in children aged 6–59 months**					
**Sample size (*n*)**	Individuals	409	350	161	199
	Households	149	131	52	50
	Sentinel sites	6	6	2	2
	Mean/sentinel site (range)	68 (24–99)	58 (21–91)	81 (75–86)	100 (83–116)
***P.falciparum***	Prevalence (95% CI)	73.8 (68.6–78.5)	62.5 (56.4–68.2)	76.0 (67.4–83.0)	72.8 (66.5–78.3)
	Range	37.3–96.2	55.5–79.7	59.3–94.7	70.3–75.8
	*p*-value		0.001		0.530
**Anemia**	Prevalence (95% CI)	85.8 (82.0–88.8)	80.0 (75.3–84.0)	87.5 (80.9–92.0)	75.9 (69.5–81.3)
	Range	67.9–92.6	67.3–89.0	82.4–93.5	68.9–85.6
	*p*-value		0.033		0.005
**Use of insecticide treated nets**	Prevalence (95% CI)	94.6 (90.9–96.8)	55.1 (47.0–63.0)	96.8 (88.0–99.2)	67.1 (53.9–78.1)
	Range	83.8–100.0	45.8–60.2	93.9–100.0	44.8–88.8
	*p*-value		<0.001		<0.001
**Indicators in children aged 10–15 years**					
**Sample size (*n*)**	Individuals	180	181	60	60
	Sentinel sites	6	6	2	2
	Mean/sentinel site (range)	30 (30–30)	30 (30–30)	30 (30–30)	30 (30–30)
***S*. *mansoni***	Prevalence (95% CI)	1.7 (0.0–3.6)	3.9 (1.0–6.7)	5.0 (0.0–10.7)	8.3 (1.1–15.5)
	Range	0.0–3.3	0.0–13.3	3.3–6.7	6.7–10.0
	*p*-value		0.203		0.464
***A*. *lumbricoides***	Prevalence (95% CI)	1.1 (0.0–2.7)	11.1 (6.4–15.6)	1.7 (0.0–5.0)	1.7 (0.0–5.0)
	Range	0.0–3.3	3.3–40.0	0.0–3.3	0.0–3.3
	*p*-value		<0.001		1.000
**Hookworm**	Prevalence (95% CI)	23.9 (17.6–30.2)	28.7 (22.1–35.4)	40.0 (27.2–52.8)	41.7 (28.8–54.5)
	Range	3.3–50.0	10.0–63.3	33.3–46.7	36.7–46.7
	*p*-value		0.296		0.853
**Indicators in children aged 6–59 months**					
***T*. *trichiura***	Prevalence (95% CI)	2.2 (0.1–4.4)	1.1 (0.0–2.6)	1.7 (0.0–5.0)	0.0 (0.0–0.0)
	Range	0.0–3.3	0.0–6.7	0.0–3.3	0.0–0.0
	*p*-value		0.406		0.315
**Indicators in women aged 15–49 years**					
**Sample size (*n*)**	Individuals	284	255	113	122
	Sentinel sites	6	6	2	2
	Mean/sentinel site (range)	47 (20–76)	43 (21–58)	57 (52–61)	61 (58–64)
**Anemia**	Prevalence (95% CI)	72.8 (67.2–77.8)	40.5 (34.4–46.8)	75.9 (68.4–82.0)	33.4 (25.5–42.5)
	Range	53.8–84.0	30.4–48.0	70.2–80.3	22.3–43.6
	*p*-value		<0.001		<0.001

### 3.3. Anemia and P. falciparum Prevalence in Children Aged 6–59 Months

Summary statistics for anemia and *P. falciparum* prevalence in children aged 6–59 months at impacted and control sites are shown in Table 2. Variation among sites is illustrated in Figure 4D,E. Significant decreases in the prevalence of anemia were observed at both impacted and control sites (both *p* < 0.05; Table 2). The prevalence *of P. falciparum* infection had decreased significantly at the impacted sites (73.8% in 2010 and 62.5% in 2013; *p* = 0.001) and non-significantly at the control sites (76.0% in 2010 and 72.8% in 2013; *p* = 0.530).

The use of insecticide-treated nets (ITNs) in six- to 59-month-old children decreased significantly at both impacted and control sites (*p* < 0.001; Table 2); specifically, from >80% in 2010 to <60% in 2013.

### 3.4. Anemia and Place of Delivery of the Last Born Child in Women Aged 15–49 Years

**Figure 5:**
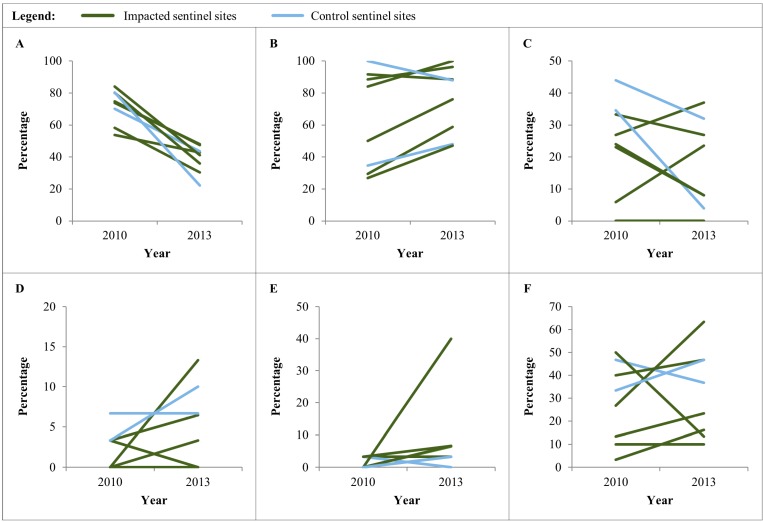
Indicators in women of reproductive age (15–49 years) and school-going children aged 10–15 years in the ABSL study area, stratified by sentinel site, 2010 and 2013. (**A**) Prevalence of anemia in women aged 15–49 years (Hb < 11 g/dL in pregnant women; Hb < 12 g/dL in non-pregnant women); (**B**) proportion of women aged 15–49 years who delivered the last child at a health facility; (**C**) proportion of women aged 15–49 years reporting that their household has an improved sanitation facility; (**D**) prevalence of *S*. *mansoni* in children aged 10–15 years; (**E**) prevalence of *A*. *lumbricoides* in children aged 10–15 years; and (**F**) prevalence of hookworm in children aged 10–15 years.

Anemia and place of delivery of the last born child in females of reproductive age were assessed as key indicators for maternal health. There was a significant reduction of anemia in women from both the impacted and the control sites (both *p* < 0.001; Table 2 and Figure 5A).

Women were asked, in the accompanying questionnaire survey, about the place of delivery of the last born child (Table 3 and Figure 5B). Since 2010, there was a significant increase in the proportion of women who gave birth in a formal healthcare facility at the impacted sites (*p* < 0.001), compared to a non-significant increase at the control sites (*p* = 0.886).

**Table 3:** Percentage of mothers giving birth at a health facility and the percentage of households having access to safe sanitation, in the ABSL study area, 2010 and 2013.

		Impacted Sentinel Sites		Control Sentinel Sites	
		2010	2013	2010	2013
**Indicators in mothers/households participating in the questionnaire survey**					
**Sample size (*n*)**	Individuals/households	144	137	51	50
	Sentinel sites	6	6	2	2
	Mean/sentinel site (range)	24 (15–26)	23 (17–27)	25 (25–26)	25 (25–25)
**% mothers giving birth at a health facility**	Prevalence (95% CI)	63.2 (54.8–71.1)	81.0 (73.4–87.2)	66.7 (52.1–79.2)	68.0 (53.3–80.5)
	Range	26.9–91.7	47.1–100.0	34.6–100.0	48.0–88.0
	*p*-value		<0.001		0.886
**% households having access to safe sanitation**	Prevalence (95% CI)	19.4 (13.3–26.9)	18.3 (12.2–25.7)	39.2 (25.8–53.9)	18.0 (8.6–31.4)
	Range	0.0–33.3	0.0–37.0	34.6–44.0	4.0–32.0
	*p*-value		0.798		0.019

### 3.5. Helminth Infection among School-Age Children and Household’s Access to Improved Sanitation

Figure 5D–F show trends in *S*. *mansoni*, *A*. *lumbricoides*, and hookworm infection rates in school-going children. A localized significant increase was found in *A*. *lumbricoides* prevalence at one impacted site (Figure 5E), being responsible for an overall increase from 1.1% in 2010 to 11.1% in 2013 (*p* < 0.001; Table 2). *S*. *mansoni*, *A*. *lumbricoides*, and *T. trichiura* infection prevalences were of low and hookworm of moderate endemicity at both baseline and the three-year follow-up.

The household questionnaire included questions regarding access to improved sanitation, which was low at both impacted and control sites (18.3% and 18.0%, respectively; Figure 5C and Table 3). There was a non-significant decrease in access to improved sanitation at the impacted sites (19.4% in 2010 and 18.3% in 2013; *p* = 0.798) and a significant decrease at control sites (39.2% in 2010 and 18.0% in 2013; *p* = 0.019).

### 3.6. Presence/Absence of Coliform Bacteria in Drinking Water Samples

Overall, no significant changes in drinking water quality in the study area were observed (Table 4). There was a non-significant increase in the proportion of household water samples contaminated with coliform bacteria from 98.6% in 2010 to 100.0% in 2013 at impacted and from 95.8% to 100.0% at control sites (*p* = 0.359 and *p* = 0.356, respectively). There was a non-significant decrease in coliform-positive samples from sources at the impacted sites (from 94.4% in 2010 to 87.5% in 2013) and a non-significant increase at the control sites (from 83.3% in 2010 to 88.9% in 2013; *p* = 0.476 and *p* = 0.756, respectively).

**Table 4:** Percentage of drinking water samples at the household and source level positive for coliform, in the ABSL study area, 2010 and 2013.

		Impacted Sentinel Sites		Control Sentinel Sites	
		2010	2013	2010	2013
**Indicators for drinking water quality at household and source level**					
**Sample size (*n*)**	Households	72	60	24	20
	Sources	18	16	6	9
	Sentinel sites	6	6	2	2
**% of household samples positive for coliform**	Prevalence (95% CI)	98.6 (92.5–99.9)	100.0 (83.2–100.0)	95.8 (78.9–99.9)	100.0 (94.0–100.0)
	Range	91.7–100.0	100.0–100.0	91.7–100.0	100.0–100.0
	*p*-value		0.359		0.356
**% of source samples positive for coliform**	Prevalence (95% CI)	94.4 (72.7–99.9)	87.5 (61.7–98.4)	83.3 (35.9–99.6)	88.9 (51.8–99.7)
	Range	50.0–100.0	66.7–100.0	75.0–100.0	75.0–100.0
	*p*-value		0.476		0.756

## 4. Discussion

### 4.1. Changes in Health Patterns

The results of the 2013 follow-up health survey revealed an improvement in most of the measured health indicators over the past three years at both impacted and control sites of the ABSL project. A multitude of contextual factors, such as deworming campaigns, distribution of ITNs by the national malaria control program, the Free Health Care Initiative, and employment seeking migration (see Figure 1), may have influenced these findings and must be taken into consideration for interpreting our data. For example, the Free Health Care Initiative launched in April, 2010, which provides pregnant women, lactating mothers and children under five years of age with free healthcare, may have contributed to positive health trends at both impacted and control sites [40]. In fact, the MoHS documented improved uptake of healthcare services in the target group in 2011, although infrastructural, drug supply, and human resource challenges continue [24,41]. The overall improvement was illustrated by health outcomes that have significantly improved at both the impacted and control sites: (i) being underweight in children under five years of age; (ii) the prevalence of anemia in children aged 6–59 months; and (iii) the prevalence of anemia in women of reproductive age.

In terms of potential project-related impacts, a set of statistically significant positive changes were observed at the impacted but not at the control sites: (i) the prevalence of stunting and wasting in children under the age of five years; (ii) *P. falciparum* prevalence in children aged 6–59 months; and (iii) the proportion of women having delivered their last born child at a healthcare facility. On the other hand, the prevalence of *A*. *lumbricoides* infection in school-age children showed a significant increase at impacted, but not control, sites.

Wasting in children under five years of age was at 2.8% at the impacted and 4.2% at the control sites in December 2013, at the beginning of the dry season. Seasonal fluctuations are expected to influence acute malnutrition, which is noticeably worse during the rainy season [37,42,43]. Hence, comparison to other surveys, such as the DHS, carried out during the rainy season, is difficult, due to the temporal heterogeneity of the surveys. Similarly, it is challenging to quantify the impact of the community-based management of acute malnutrition (implemented since 2007) or other programs [26].

Stunting, an indicator for chronic malnutrition (32.1% at the impacted and 40.7% at the control sites), is associated with a number of immediate factors (e.g., environmental, economic, and sociopolitical factors restricting access to safe and sufficient food and water) and underlying causes (e.g., inadequate care, limited access to health services, and household food security), with poverty being an overarching determinant for each of these [44].

The significant decrease in wasting and stunting at the impacted compared to the non-significant decrease at the control sites over the three-year period potentially reflects: (i) the ABSL farmer development programs initiated in the ABSL project area (see Figure 1); (ii) people’s increased ability to access food, healthcare, and other essential commodities in the ABSL project area; and (iii) the in-migration of children from areas with lower rates of stunting or wasting into the area since 2010.

The prevalence of *P. falciparum* has declined in the study population between 2010 and 2013. Possible contributors include: (i) a decrease in disease transmission due to interventions, such as ITN distribution (in 2010) and focal indoor residual spraying campaigns [23,25,45,46]; (ii) the improved diagnostic capacity using RDTs; (iii) an increased availability (including accessibility and affordability) of ACTs in medicine outlets [24,40,47]; (iv) environmental changes (e.g., change of vegetation, urbanization, and alteration of breeding sites) [48,49,50]; and (v) an increased awareness of, and improved economic conditions to, utilize protective measures [51]. The national mass-distribution campaign of ITNs was ongoing at the time of the BHS, and a study six months after the distribution found 87.6% of households owned at least one ITN, with 76.5% of households regularly sleeping under an ITN [45]. As per our findings in December 2013, ITN possession decreased to 55.1% at the impacted and 67.1% at the control sites. Reduced ITN coverage is expected over time, as these nets get destroyed, and repeated mass-distributions are therefore needed to maintain and extend coverage [52]. In Sierra Leone, another nation-wide distribution took place in June 2014 [53]. In the study area, the lower rate at the impacted compared to the control sites might be due to the fact that people have migrated into the ABSL project area after the 2010 campaign [17]. Despite the overall lower ITN coverage, *P. falciparum* prevalence among children aged 6–59 months has decreased significantly at the impacted sites, suggesting that the previously-mentioned health system, environmental, and economic changes are at play. Still, two in three children were found to be infected with *P. falciparum*, calling for sustained efforts in vector control and malaria management in the study area.

Despite focal increases in the prevalence rates of helminth infections, most changes were not significant and corresponded with spatial predictions [54]. The significant increase in *A*. *lumbricoides* can be attributed to a small cluster of children in Masetheleh, a village without a health facility, that had not been effectively reached by the national deworming program [55]. The changes in the prevalence of *S*. *mansoni* were attributed to children who had migrated from highly endemic areas into the study area, as confirmed by children and teacher interviews. As per Figure 1, national deworming campaigns by the MoHS and restoration of wells initiated by the ABSL project in the area since 2012 should contribute to helminth control. However, the control of helminth infections and other soil- and water-related diseases is only possible if environmental sanitation conditions are extensively improved, complemented with increased access to safe drinking water and behavior change [56,57]. Currently, less than 20% of the households at both impacted and control sites have access to improved sanitation, and over 80% drink fecally contaminated water. The data suggest that project developments have not translated into improved water and sanitation indicators, partly because communities’ demands and capacities to take action on their own (e.g., resources and technical expertise) are limited [58,59]. The IFC’s performance standards require ABSL to mitigate potential impacts related to its activities and encourage corporate social investment [21,60]. Project-related in-migration bears the risk of worsening the water and sanitation situation in the project area [61]. Thus, it is recommended that ABSL sets water and sanitation-oriented interventions, along with health system strengthening as priorities for corporate social investment, also because such efforts are urgently needed for combating diarrheal and other infectious diseases, including the current Ebola outbreak in West Africa [62].

Anemia serves as an indicator for the general wellbeing of a child, since it is a multi-factorial condition governed by malnutrition, malaria, hookworm, and *Schistosoma* infections, hereditary hemoglobinopathies, and poor socioeconomic status [63]. The significant reduction of anemia at both impacted and control sites might indicate a general improvement of child health in the study area. In 2013, anemia prevalences in children at the impacted (80.0%) and control sites (75.9%) were slightly lower than the Northern region average found in the 2013 DHS (83.4%) [37]. A meaningful reduction of anemia in children requires the reduction of the overall disease burden, an increased individual awareness and capacity to tackle the underlying causes (e.g. investment in protection against parasites), and an understanding of the contribution of hemoglobinopathies.

Maternal health indicators in the study population, anemia in women of reproductive age, and the proportion of deliveries in health facilities had improved since the BHS. Project-induced development of roads might have facilitated accessibility to healthcare structures, as there was a more pronounced increase of the proportion of deliveries at a health facility at impacted compared to control sites [8]. Nevertheless, the increase at the control sites might suggest a cumulative impact of improved road infrastructure, as well as increased levels of income in the study area. In Sierra Leonean healthcare facilities, an increase of 45% of facility-based deliveries was noted in the first 12 months of the Free Health Care Initiative compared to the preceding 12 months, indicating that it was an important factor [24].

### 4.2. Implications for the Health Impact Assessment (HIA) Process

The three-year follow-up health survey was conducted as an integral part of the HIA, the overall environmental and social monitoring program, and the company’s community health and safety management plan. The described study methodology presents a feasible approach for health monitoring in settings where routine health information systems might not reliably pick up the subtle changes at the community level over time [64,65]. The present study is among a few rigorous monitoring approaches based on repeated cross-sectional surveys in sub-Saharan Africa that provide evidence on how a large infrastructure project impacts community health. The baseline data collected provided an indispensable benchmark for monitoring changes in health patterns over time [22]. By conducting repeated cross-sectional surveys, ABSL has created an evidence-base for decision-making and prioritization of its health mitigation measures.

Besides the evidenced-based decision-making and critical monitoring of changing health patterns at impacted sites, the measurement of health outcomes assists the evaluation of the HIA, in particular the prediction accuracy of health impacts made in the risk assessment phase. In fact, process evaluation (*i.e*., the process leading to a decision on health interventions) and the outcome evaluation (*i.e.*, the outcome of a health intervention) are both important steps to improve the HIA as a decision-support tool, and epidemiological case studies represent an essential tool for the latter [66]. Moreover, the HIA and associated research gain transparency, value, and credibility by undergoing a peer-reviewed process [65].

### 4.3. Limitations of the Study

The findings are specific for the selected sentinel sites and not representative for a wider area. The type and duration of ABSL impacts may vary among these, since the construction phase of the project was ongoing from 2010 to 2014, meaning that infrastructural and community developments, including road constructions, planting of sugarcane fields, and implementation of the farmer development program, were gradually introduced into the ABSL area [16,17]. To minimize seasonal effects, the BHS and the three-year follow-up study were conducted at the same time of year, but fluctuations due to annual climate variations might still influence health indicators. The study did not control for the in- or out-migration characteristics of the study participants. The diagnosis of helminth infections using the Kato–Katz method with only one specimen and one urine centrifugation has a lower sensitivity than multi-specimen Kato–Katz and concentration methods, and the reported values might be underestimated [67]. Although the DelAgua^®^ testing kit used in 2013 allowed for a precise coliform count and, thus, categorization according to WHO standards (samples with 0–10 coliforms/100 mL are considered as tolerable to drink without prior treatment), only the absence/presence categories were applied here, in order to allow for direct comparison [68].

## 5. Conclusions

The findings of two cross-sectional health surveys, conducted exactly three years apart, show a significant decrease in the prevalence of stunting, wasting, and *P. falciparum* in children under five years of age and a significant increase in the proportion of women having delivered their last child in a healthcare facility at impacted sites, which was not seen at the control sites. The prevalences of being underweight and anemia in children and anemia in women have significantly decreased at both impacted and control sites. Access to improved sanitation decreased significantly at control and non-significantly at impacted sites. Fecal contamination of drinking water at both the source and household level had not changed significantly and, indeed, remained unacceptably high.

Overall, much remains to be done to further improve the overall health and wellbeing of the population in the study area. Periodic follow-up health surveys, as part of the HIA, deepen the understanding of changing health patterns in local communities and allow for adaptations in the community health and safety management plan of the ABSL, including specific health interventions. Since scientific evidence and its ethical use is one of the core values for good practice in HIA [69], the approach of using independent, evidence-based research that allows for an objective and transparent evaluation of changing health patterns could set an example for similar projects elsewhere in sub-Saharan Africa and other low- and middle-income countries in Asia and Latin America [16,22].
